# Beyond traditional criteria: CRP and fibrinogen as biomarkers of metabolic syndrome in ART-treated people living with HIV and their association with *HIF-1α* rs11549465

**DOI:** 10.1371/journal.pone.0357899

**Published:** 2026-09-08

**Authors:** Nemanja Đorđević, Jovan Ranin, Ivana Gmizić, Biljana Ljujić, Marko Marković, Ivan Rajković, Jovana Ranin, Ivana Raković, Sofija Sekulić Marković, Biljana Popovska Jovičić

**Affiliations:** 1 Department of Infectious Diseases, Faculty of Medical Sciences, University of Kragujevac, Kragujevac, Serbia; 2 Clinic for Infectious Diseases, University Clinical Centre Kragujevac, Kragujevac, Serbia; 3 Department of Infectious Diseases, Faculty of Medicine, University of Belgrade, Belgrade, Serbia; 4 Clinic for Infectious and Tropical Diseases, University Clinical Centre of Serbia, Belgrade, Serbia; 5 Department of Genetics, Faculty of Medical Sciences, University of Kragujevac, Kragujevac, Serbia; University of Texas Medical Branch at Galveston, UNITED STATES OF AMERICA

## Abstract

Metabolic syndrome (MS) is highly prevalent among people living with HIV (PLWH) receiving antiretroviral therapy (ART) and is driven by persistent low-grade inflammation. Hypoxia-inducible factor 1 alpha (*HIF-1α*) polymorphisms may influence inflammatory pathways underlying MS. This study investigated the association of the rs11549465 *HIF-1α* polymorphism with inflammatory markers and MS in PLWH receiving ART. We conducted a multicenter case-control study including 116 PLWH treated at two University Clinical Centers in Serbia. Participants were classified according to NCEP ATP III criteria. Genotyping was performed by Real-Time PCR using TaqMan assays. C-reactive protein (CRP) and fibrinogen levels were significantly higher in participants with MS (p < 0.05). ROC analysis demonstrated their potential as biomarkers of MS with cut-off values of 1.85 mg/L for CRP and 2.49 g/L for fibrinogen. Age significantly correlated with CRP and Neutrophil/Lymphocyte ratio (p < 0.05). Elevated CRP and fibrinogen levels were associated with hypertension, hypertriglyceridemia, and other MS-abnormalities (p < 0.05). The *HIF-1α* rs11549465 CT + TT genotype was associated with higher IL-6 levels among participants with MS (p = 0.026). CRP and fibrinogen may complement existing MS criteria in identifying PLWH at increased risk of cardiovascular disease and diabetes. These findings highlight the role of chronic inflammation and genetic variability in metabolic complications of HIV.

## 1. Introduction

Metabolic syndrome (MS) represents a cluster of interrelated metabolic abnormalities that significantly increase the risk of cardiovascular disease (CVD) and type 2 diabetes mellitus (DM) [1]. Its prevalence is higher among people living with HIV (PLWH) receiving antiretroviral therapy (ART) compared to the general population, reflecting the combined effects of chronic infection, long-term ART exposure, and persistent immune activation [2,3].

Chronic low-grade inflammation is recognized as a key driver of metabolic disturbances in PLWH [4]. Often referred to as meta-inflammation, this process is closely linked to visceral obesity and is considered a key pathophysiological mechanism underlying MS. Adipose tissue acts as an active endocrine organ, producing numerous proinflammatory mediators, including interleukins (IL-1β, IL-6, TNF-α), and acute-phase proteins such as C-reactive protein (CRP) and fibrinogen [5,6]. In PLWH, persistent immune activation and residual inflammation remain evident despite effective antiretroviral therapy (ART) and viral suppression. Several studies have demonstrated elevated circulating inflammatory markers compared to HIV-negative individuals, reflecting ongoing immune dysregulation [7]. Cohort studies, including the FRAM study, have linked increased fibrinogen and CRP levels with altered adipose distribution and metabolic abnormalities in HIV infection [8]. Likewise, elevated interleukin 6 (IL-6), CRP, fibrinogen and D-dimer have been associated with increased cardiometabolic risk and mortality in this population, consistent with findings in the general population [9–11].

Hypoxia-inducible factor-1 alpha (HIF-1α), encoded by the *HIF-1α* gene, is a key regulator of cellular adaptation to hypoxia and plays an important role in metabolic and inflammatory pathways [12]. The *HIF-1α* rs11549465 (C > T, Pro582Ser) polymorphism is a functional variant linked with enhanced HIF-1α activity [13,14]. While this polymorphism has been linked to MS and related conditions in general population [15], data in PLWH are lacking. Mechanistically, HIF-1α promotes cytokine transcription (e.g., IL-6, VEGF), linking hypoxia to chronic inflammation, which can be practically assessed via circulating biomarkers [9,16]. Among them, CRP-particularly high-sensitivity CRP (hs-CRP)- and fibrinogen are of particular interest due to their availability and established association with cardiometabolic risk [17,18]. However, their role as clinically applicable biomarkers for identifying MS in ART-treated PLWH remains insufficiently defined.

The aim of this study evaluates CRP and fibrinogen as accessible MS biomarkers in PLWH on ART, exploring their links to inflammatory pathways and the *HIF-1α* rs11549465 polymorphism. By focusing on widely available serum markers, we provide clinically applicable insights into chronic inflammation, genetic variability, and metabolic risk in HIV. While prior research on this cohort investigated the *HIF-1α* polymorphism in relation to MS, this study offers a distinct analytical focus by specifically evaluating inflammatory biomarkers [19].

## 2. Materials and methods

### 2.1. Study population

We conducted a multicenter, cross-sectional study including 116 PLWH, both outpatients and hospitalized individuals, treated at the Clinic for Infectious Diseases at University Clinical Center Kragujevac (UKCKg), and the Clinic for Infectious and Tropical Diseases, University Clinical Center of Serbia (UKCS). Eligible participants were adults (≥18 years) of Caucasian ethnicity receiving ART for at least 18 months [20]. HIV infection was confirmed by reverse transcriptase polymerase chain reaction (RT-PCR). Individuals with MS at the time of HIV diagnosis and ART initiation were excluded. Additional exclusion criteria included pregnancy and breastfeeding. Patients with acute infections, as well as those experiencing an exacerbation of chronic non-infectious diseases or conditions, were excluded from the study. Additionally, individuals with critical illnesses or those in the postoperative phase were not included.

All participants provided written informed consent. The study was conducted in accordance with the Declaration of Helsinki and Good Clinical Practice guidelines, and was approved by the Ethics Committees of UKCKg, the Faculty of Medical Sciences, University of Kragujevac (FMNKg), and UKCS (approval numbers 01/22–38, 01–3253, and 307/31, respectively).

### 2.2. Data collection

During a single routine outpatient visit (index visit), anthropometric and clinical assessments were conducted, and blood samples were collected for laboratory testing. Data concerning demographics and HIV infection were retrospectively obtained from the hospital’s information system and linked to the patient’s initial visit. This data included age, sex, duration of HIV infection, ART regimen and duration, HIV RNA levels, anthropometric measurements and relevant laboratory parameters. Participants were required to have been on ART without interruption for a minimum of 18 months by the time of the study assessment to qualify. Those who had not yet achieved this duration were reassessed during a later routine visit once they met the eligibility criteria. PLWH who did not have a valid index visit, such as those who ceased ART before completing 18 months, were not included in the study. The investigation was carried out over the period from April 2023 through December 2024.

The presence of hypertension (HTA), dyslipidemia, and DM, as well as ongoing treatment for these conditions, was assessed at enrollment. Patients receiving antihypertensive, lipid-lowering, or antidiabetic treatment were classified accordingly, regardless of current laboratory values. Anthropometric measurements included body weight, height, and waist circumference, were measured using standardized procedures. Body mass index (BMI) was calculated as weight in kilograms divided by height in meters squared (kg / m²). Blood pressure was measured after a 15-minute rest period using a calibrated aneroid sphygmomanometer.

Following an overnight fast of at least 12 hours, venous blood samples were collected for biochemical analysis using a Vacutainer system. An 8 mL blood sample was collected in a serum separation tube (SST) for biochemical analysis, while two more 5 mL samples were taken in ethylenediaminetetraacetic acid (EDTA) tubes for the purposes of complete blood count determination and HIV RNA quantification. Lipid profile (total cholesterol (TC), low-density lipoprotein cholesterol (LDL-C), high-density lipoprotein cholesterol (HDL-C), triglycerides (TG)), and fasting glucose were determined, along with inflammatory markers including CRP, fibrinogen, D-dimer, and ferritin. Complete blood count was used to calculate the N/L ratio, and HIV RNA levels were measured by RT-PCR. All laboratory analyses were performed using standardized protocols in the central laboratory of UKCKg.

Participants were categorized according to the National Cholesterol Education Program Adult Treatment Panel III (NCEP ATP III) criteria [21]. MS was defined as the presence of at least three of the following components: abdominal obesity, elevated TG, reduced HDL-C, elevated BP, and increased fasting glucose. Participants not meeting these criteria were classified as controls.

### 2.3. DNA extraction and SNP genotyping

Genomic DNA was extracted from 200 µL of whole blood collected in EDTA tubes using the Thermo Scientific GeneJET Genomic DNA Purification Kit, according to the manufacturer’s instructions. Genotyping of the the *HIF-1α* gene polymorphism rs11549465 polymorphism was performed using a TaqMan® SNP Genotyping Assay (assay ID: C__25473074_10) by real-time PCR on a Bio-Rad CFX96 Real Time PCR (Bio-Rad, USA). All genotyping results were independently evaluated by two researchers, with complete concordance observed in in repeated analyses.

### 2.4. Statistical analysis

Sample size (n = 116) was estimated using G*Power version 3.1, assuming an effect size of 0.3, a type I error (α) of 0.05, and a statistical power of 80% for df = 1. Data analysis was performed in SPSS version 26 (IBM, Armonk, NY, USA). The Hardy-Winberg equilibrium for genotype distribution was assessed using an exact test (Guo & Thompson) due to low frequency of *HIF-1α* rs11549465 TT genotype. As most variables were not normally distributed, nonparametric tests were applied, including the Mann–Whitney U test and Kruskal-Wallis test for continuous variables, and the chi-square test or Fisher’s exact test for categorical variables, as appropriate. Association between variables were accessed using Spearman correlation coefficients. Simple linear regression analysis was performed for selected variables demonstrating significant correlations. Receiver operating characteristic (ROC) curve analysis was used to assess the discriminatory ability of CRP and fibrinogen for MS. The area under the curve (AUC) was used to quantify predictive performance, and optimal cut-off values were determined using the Youden index. Sensitivity, specificity, and corresponding 95% confidence intervals were calculated. Based on these cut-off values, biomarkers were dichotomized for further analysis. Multivariable logistic regression model with enter method was performed to assess independent associations of CRP and fibrinogen with MS, adjusting for age, ART duration, ART regimen group, and *HIF1α* genotype. Covariates were chosen based on their individual associations with MS, biological significance, and existing evidence. Components defining MS were excluded as covariates to prevent overadjustment. Model assumptions were assessed prior to analysis. No relevant multicollinearity was observed (variance inflation factor <1.5). Linearity of the logit was confirmed for continuous predictors using the Box–Tidwell approach, while fibrinogen was categorized into terciles due to non-linearity. The model’s fit was checked by using the Hosmer–Lemeshow goodness-of-fit test. Odds ratios (ORs) with 95% confidence intervals (CIs) were reported. A p-value <0.05 was considered statistically significant.

## 3. Results

### 3.1. General characteristics

The prevalence of MS in the study population was 38.8% The median age of participants was 44 years (IQR 39–53.5; range 23–78), while the median duration of HIV and ART was 10 years (IQR 5–15; range 2–31). No statistically significant differences were observed in inflammatory markers between participants stratified by age, duration of HIV, or duration of ART (p > 0.05). When comparing inflammatory markers between genders, only ferritin levels were significantly higher in men (p < 0.001), with no significant differences observed for other markers. Table 1 shows demographic, HIV-related, cardiometabolic and laboratory characteristics, as well as differences in inflammatory parameters between PLWH with MS and controls. Participants with MS had a longer duration of HIV infection and ART exposure (p < 0.05). All participants had undetectable viremia, and their immune status was generally stable. Significantly higher values of systolic blood pressure (SBP), diastolic blood pressure (DBP), BMI, TG and glycemia were observed in participants with MS (p < 0.001), along with lower HDL-C levels (p < 0.05). Among inflammatory markers, only CRP and fibrinogen levels were significantly higher in the MS group (p < 0.05).

**Table 1:** Demographic and clinical features.

			Metabolic syndrome n=45/116 (38.8%)	Controls n=71/116 (61.2%)	*p*
**Demographic and HIV-related characteristics**					
Age (years)			53 (45.00-60.5)	41 (37-46)	***
Gender	Males, n (%)		33 (73.3%) 12 (26.7 %)	60 (84.5 %) 11 (15.5 %)	ns^†^
	Females, n (%)				
HIV duration (years)			12 (6.5-16.5)	9 (5-15)	*
ART duration (years)			11 (6.5-16.0)	8 (5-14)	*
**Cardiometabolic profile**					
SBP ^‡^ (mmHg)		140 (127.5-148)		120 (115-130)	***
DBP^‡^ (mmHg)		80 (80-90)		79 (70-80)	***
BMI (kg/m²)		26.42 (24.76-29.05)		23.89 (22.00-25.83)	***
TC ^‡^ (mmol/l)		5.24 (4.41-5.88)		5.0 (4.33-5.36)	ns
LDL- C^‡^ (mmol/l)		2.89 (2.16-3.67)		2.98 (2.49-3.58)	ns
HDL- C^‡^ (mmol/l)		1.2 (1.02-1.50)		1.28 (1.18-1.64)	*
TG^‡^ (mmol/l)		1.81 (1.10-2.55)		1.05 (0.74-1.40)	***
Glycemia^‡^ (mmol/l)		5.7 (5.05-6.35)		5.2 (4.90-5.40)	***
**Inflammatory parameters**					
CRP (mg/L)		2.05 (0.98-3.90)		1.10 (0.50-1.88)	**
Fibrinogen (g/L)		2.70 (2.50-3.16)		2.50 (2.28-3.0)	*
D-dimer (mg/L)		0.30 (0.20-0.32)		0.27 (0.19-0.39)	ns
IL-6 (pg/mL)		2.6 (1.5-3.7)		2.2 (1.7-3.8)	ns
Ferritin (ug/L)		128.2 (52.9-268.5)		86.0 (59.0-178.0)	ns
N/L ratio		1.76 (1.21-2.11)		1.56 (1.25-2.31)	ns

Values are presented as median (IQR) unless otherwise indicated. Categorical variables are expressed as number (n) and percentage (%). Abbreviations: HIV – Human immunodeficiency virus; ART – Antiretroviral therapy; SBP – Systolic blood pressure; DBP – Diastolic blood pressure; BMI – Body Mass Index; TC – Total cholesterol; LDL-C – low-density lipoprotein cholesterol; HDL-C – high-density lipoprotein cholesterol; TG – triglycerides; CRP – C- reactive protein; IL-6 – Interleukin 6; N/L ratio – Neutrophil/Lymphocyte ratio. *p < 0.05; **p < 0.01; ***p < 0.001; ns = not significant. Continuous variables were compared using the Mann–Whitney U test unless otherwise indicated. Categorical variables were compared using the chi-square test or Fisher’s exact test, as appropriate. † – Pearson Chi-Square test; ‡ - Patients taking antihypertensive, lipid-lowering, and antidiabetic drugs were excluded from analysis.

### 3.2. Demographic and HIV influences and inter-marker associations within the inflammatory profile

**Fig 1:**
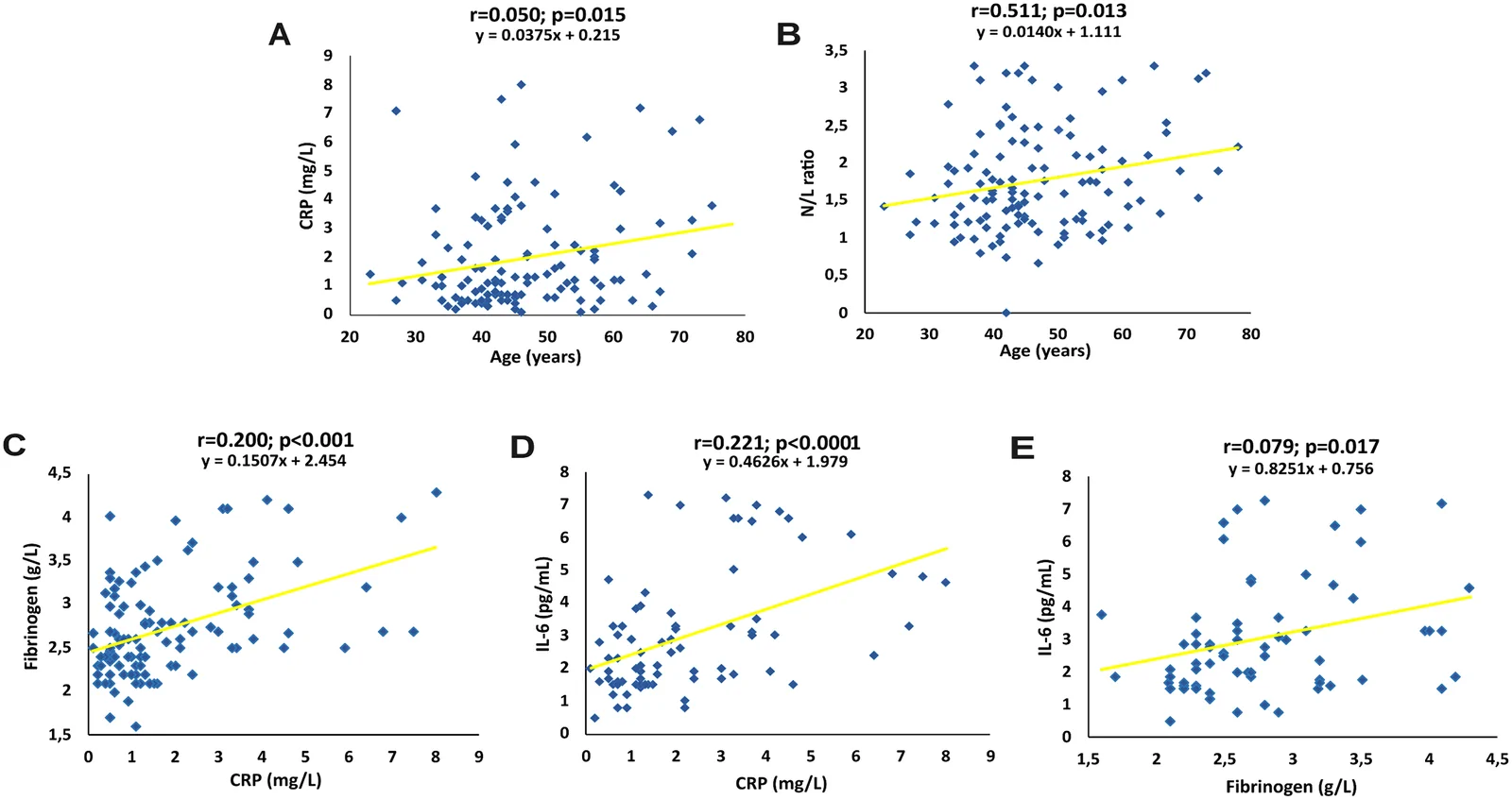
Associations between age and inflammatory markers, and interrelationships among inflammatory biomarkers. **(A–B)** Simple linear regression analyses presenting positive correlations between age and circulating C-reactive protein (CRP) levels (**A**) and the neutrophil-to-lymphocyte (N/L) ratio **(B)**. **(C–E)** Relationships among low-grade chronic inflammatory markers. CRP showed positive association with fibrinogen (**C**) and interleukin 6 (IL-6) **(D)**, while fibrinogen exhibited a weaker but significant positive correlation with IL-6 **(E)**. Each dot represents an individual participant; yellow lines indicate the linear regression fit with corresponding equations, Spearman correlation coefficients (r), and probability (*p*) values.

To investigate the relationships between demographic characteristics, HIV-related factors and inflammatory markers, we conducted simple linear regression analyses utilizing age, sex, HIV and ART duration as independent variables. Age showed a statistically significant positive correlation with CRP levels (r^2^ = 0.050; p = 0.019). A significant association was also observed between age and the N/L ratio (r^2^ = 0.511; p = 0.013) (Fig 1A-1B).

Analysis of inter-marker relationship demonstrated that CRP was positively associated with IL-6 (r^2^ = 0.221; p < 0.001), and fibrinogen (r^2^ = 0.200; p < 0.001). Additionally, fibrinogen and IL-6 exhibited a moderate yet significant correlation (r^2^ = 0.079; p = 0.019) (Fig 1C-1E).

No significant associations were observed between sex and inflammation biomarkers, except for higher ferritin levels in men, nor between age and IL-6 or fibrinogen levels. No meaningful associations were identified between inflammatory markers and other demographic or clinical variables.

### 3.3. ART characteristics

At the time of ART initiation, 43/116 (37.1%) participants were receiving non-nucleoside reverse transcriptase inhibitor (NNRTI)-based regimen, 45/116 (38.8%) protease inhibitor (PI)-based regimens, and 28/116 (24.1%) integrase strand transfer inhibitors (INSTI)-based regimens. At study entry, after a median ART duration of 10 years (IQR 5–15), the distribution had changed, with 22/116 (19.0%) on NNRTI-based therapy, 15/116 (12.9%) on PI-containing regimens, and 79/116 (68.1%) were on INSTI-based ART. Nucleoside reverse transcriptase inhibitors (NRTI) were not examined as a distinct category, as they represent the backbone of most ART regimens. Abacavir was used at some point during treatment in 57/116 (49.1%) participants. Based on current ART, unadjusted analyses showed differences in MS prevalence across regimen groups: highest in PI group (53.3%), intermediate in INSTI group (40.5%), and lowest in the NNRTI group (22.7%). However, in unadjusted logistic regression, the ART regimen was not significantly associated with MS overall (global p = 0.160). Compared to NNRTI-based therapy (reference), the PI group showed a higher, though not statistically robust, odds of MS (OR = 3.66, 95% CI 0.90–15.19; p = 0.048), while no significant association was observed for INSTI-based regimens (OR = 2.32, 95% CI 0.78–6.91; p = 0.133).

No statistically significant differences in inflammatory biomarkers were observed across ART regimen groups for CRP (p = 0.595), fibrinogen (p = 0.086), D-dimer (p = 0.892), IL-6 (p = 0.386), ferritin (p = 0.825), or N/L ratio (p = 0.304).

### 3.4. Assessment of CRP and fibrinogen levels as potential biomarkers of MS in ART-treated PLWH

**Fig 2:**
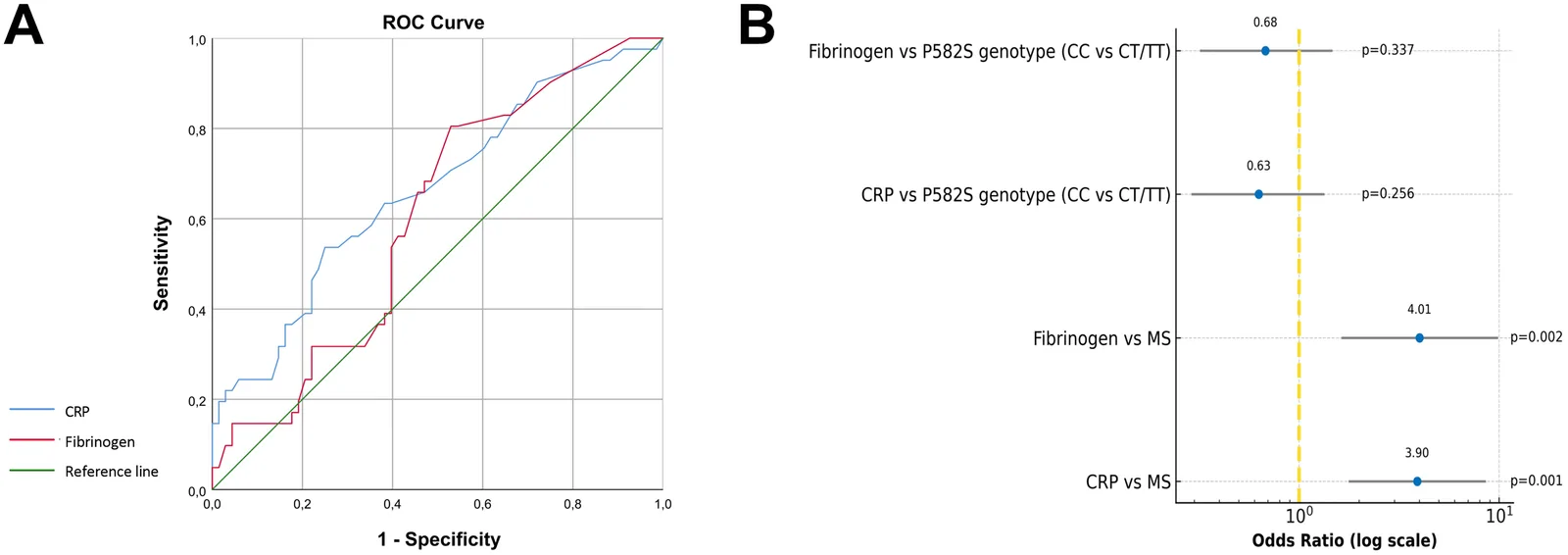
The predictive value and correlation of CRP and fibrinogen levels with MS and *HIF-1α* rs11549465 genotypes in ART-treated PLWH. **(A)** Receiver operating characteristic (ROC) curves illustrating the discriminative capacity of CRP and fibrinogen levels in predicting MS among PLWH on ART. **(B)** A forest plot showing odds ratios (OR, log scale) with 95% confidence intervals (95% CI) for the relationship between fibrinogen and CRP levels and *HIF-1α* rs11549465 (P582S) genotypes and MS using cut-off levels previously obtained by ROC curve. Dominant genetic model (CC vs CT + TT) was used because of small frequency of minor allele T. The squares show point estimates of the OR, and the horizontal bars represent the 95% CIs. The vertical line at OR = 1 is the reference point. Probability (*p*) values are presented to the right of each estimate. Due to the sample size, the 2x2 contingency tables were used for OR and 95% CI calculation, and Fisher’s exact test was used to determine *p* values.

CRP and fibrinogen were identified as the only inflammatory markers significantly elevated in participants with MS. To explore their potential as accessible serum biomarkers of MS, ROC curve analysis was performed (Fig 2A). The AUC for CRP was 0.684 (SE = 0.050, 95% CI: 0.59–0.78, p = 0.001). The optimal cut-off value, determined by the Youden index, was 1.85 mg/L, with a sensitivity of 56.5% and specificity of 75.0% (J = 0.315). Fibrinogen demonstrated an AUC of 0.612 (95% CI: 0.51–0.72, p = 0.048), with an optimal cut-off value of 2.49 g/L, yielding a sensitivity of 81% and specificity of 48.6% (J = 0.296).

Using these cut-off values, CRP and fibrinogen were further analyzed as categorical variables. Participants with MS showed significantly higher proportions of elevated CRP and fibrinogen levels compared to the control group (p < 0.01) (Fig 2B).

### 3.5. Comparison of CRP and fibrinogen levels in relation to comorbid conditions linked to MS in ART-treated PLWH

CRP and fibrinogen levels were significantly higher in the PLWH with MS compared to controls (Table 1). Further analysis assessed their distribution across MS-related comorbidities (Table 2). In the overall study population, participants with HTA and elevated TG levels had significantly higher levels of both CRP and fibrinogen (p < 0.05). Elevated CRP levels were also observed in individuals with dyslipidemia and increased BMI, while higher fibrinogen levels were associated with low HDL (p < 0.05).

**Table 2:** Distribution of CRP and fibrinogen levels across MS-related comorbidities in PLWH on ART.

Comorbidities		CRP		Fibrinogen	
		Mdn (IQR)	*p*	Mdn (IQR)	*p*
**HTA^†^**	Present	2.0 (0.7, 4.3)	**0.042**	2.70 (2.49, 3.28)	**0.047**
	Absent	1.2 (0.6, 2.3)		2.52 (2.30, 2.95)	
**Dyslipidemia**	Present	1.7 (0.7, 3.3)	**0.038**	2.70 (2.40, 3.20)	0.057
	Absent	1.1 (0.5, 2.0)		2.51 (2.20, 2.99)	
**TC**	High	1.2 (0.7, 3.0)	0.900	2.52 (2.30, 3.00)	0.942
	Normal	1.3 (0.6, 3.1)		2.60 (2.30, 3.03)	
**LDL-C**	High	1.2 (0.6, 3.1)	0.628	2.59 (2.31, 3.20)	0.881
	Normal	1.4 (0.7, 3.0)		2.67 (2.30, 2.90)	
**HDL-C^†^**	Low	1.9 (0.5, 3.4)	0.404	3.12 (2.69, 3.49)	**0.002**
	Normal	1.2 (0.7, 3.1)		2.57 (2.30, 2.91)	
**TG^†^**	High	1.9 (0.7, 4.6)	**0.046**	2.95 (2.60, 3.34)	**0.000**
	Normal	1.2 (0.6, 2.3)		2.50 (2.30, 2.88)	
**Glycemia^†^**	Elevated	1.2 (0.7, 3.2)	0.499	2.52 (2.30, 2.90)	0.357
	Normal	1.2 (0.5, 2.9)		2.69 (2.30, 3.24)	
**BMI**	Elevated	1.5 (0.8, 3.7)	**0.008**	2.70 (2.30, 3.20)	0.140
	Normal	1.1 (0.5, 2.1)		2.50 (2.30, 2.92)	

The results are expressed as median (IQR). Statistically significant results are shown in bold. Dyslipidemia was defined as the presence of at least one abnormal lipid parameter or low HDL-C. High total cholesterol was defined as > 5.2 mmol/l, and high LDL-C as > 2.59 mmol/l. The BMI cut-off value was 25 kg/m2. Abbreviations: † - defined according to NCEP ATP III criteria; CRP – C- reactive protein; HTA – Hypertension; TC – Total cholesterol; LDL-C – low-density lipoprotein cholesterol; HDL-C – high-density lipoprotein cholesterol; TG – triglycerides; BMI – Body Mass Index.

**Fig 3:**
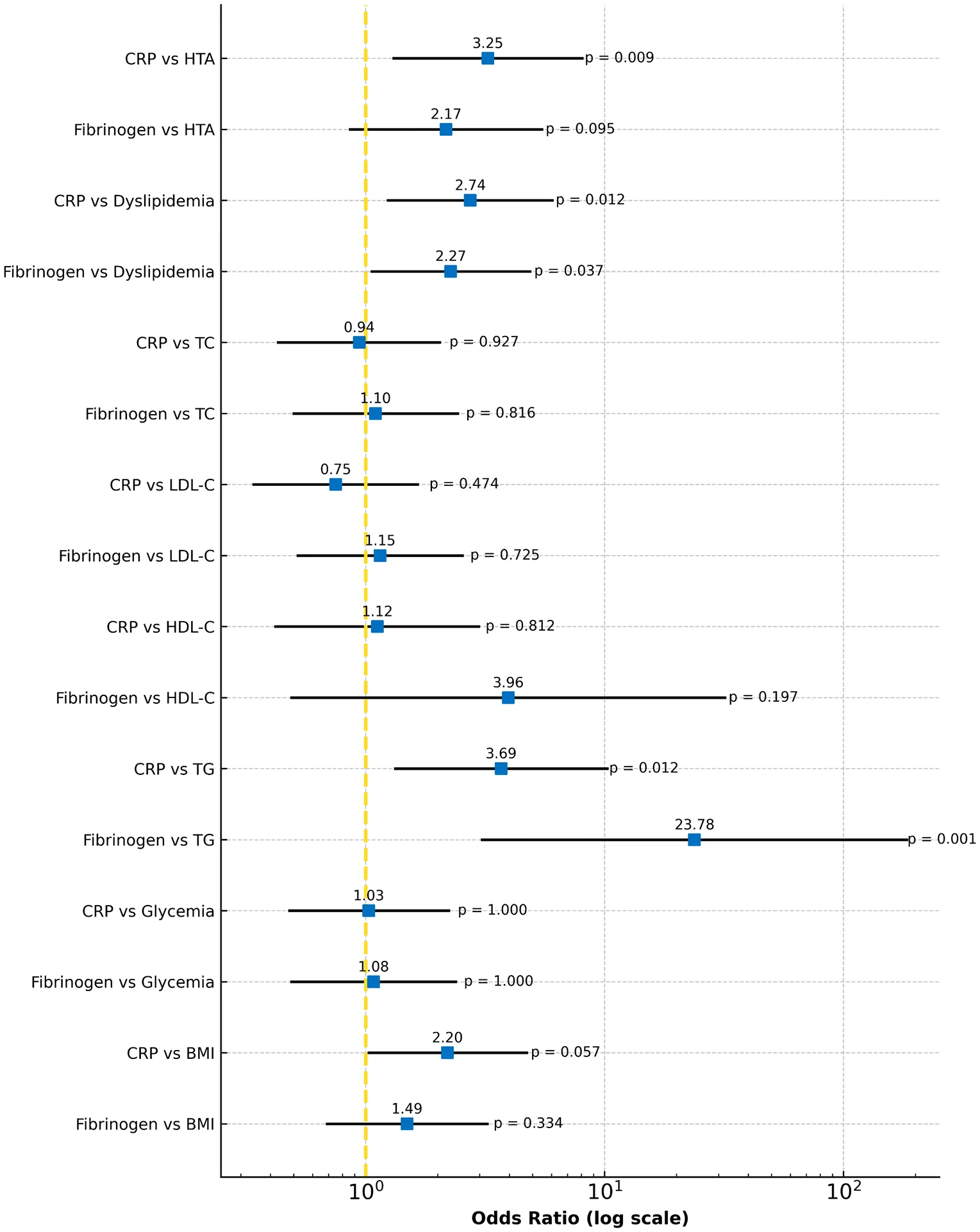
Association in CRP and fibrinogen levels with MS-related comorbidities in PLWH on ART. Forest plot showing odds ratios (ORs) with 95% confidence intervals (CIs) for the association between elevated CRP and fibrinogen levels (based on ROC-derived cut-off values) and individual components of MS (hypertension [HTA], dyslipidemia, total cholesterol [TC], LDL-C, HDL-C, triglycerides [TG], glycemia, and BMI). Squares represent point estimates, horizontal lines indicate 95% CIs, and the vertical line (OR=1) represents the reference value.

Using ROC-derived cut-off values, the discriminative capacity of CRP and fibrinogen was evaluated in relation to metabolic abnormalities (Fig 3). Elevated CRP levels were significantly associated with HTA (OR = 3.25, 95% CI = 1.33–7.93, p = 0.009), dyslipidemia (OR = 2.74, 95% CI = 1.25–6.03, p = 0.012), and elevated TG levels (OR = 3.69, 95% CI = 1.34–10.14, p = 0.012). Similarly, elevated fibrinogen levels were significantly linked to dyslipidemia (OR = 2.27, 95% CI = 1.05–4.89, p = 0.037) and elevated TG levels (OR = 23.78, 95% CI = 3.67–154.00, p = 0.001). No statistically significant association found between either biomarker and TC, LDL-C, HDL-C, glycemia, or BMI.

### 3.6. Comparison of chronic low-grade inflammation marker levels between *HIF-1α* rs11549465 genotypes in PLWH receiving ART

The genotype distribution conformed to Hardy–Weinberg equilibrium according to the exact test (p = 0.077). No statistically significant differences were observed between genotypes in the overall study population (p > 0.05).

**Fig 4:**
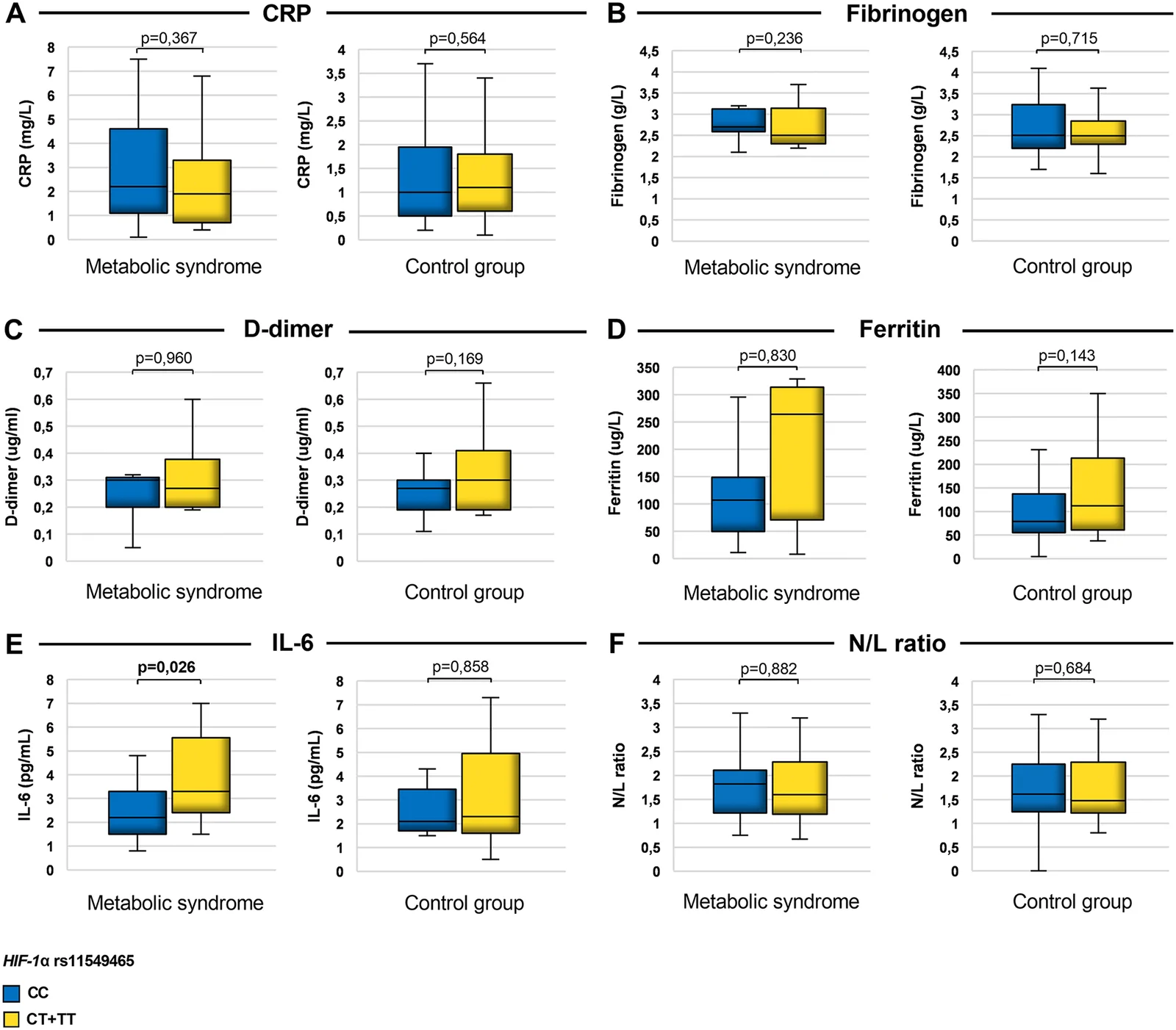
Distribution of inflammatory and coagulation biomarkers according to *HIF-1α* rs11549465 genotype in PLWH on ART with and without metabolic syndrome. Box-and-whisker plots show serum levels of **(A)** C-reactive protein (CRP), (**B**) fibrinogen, **(C)** D-dimer, (**D**) ferritin, (**E**) interleukin-6 (IL-6), and (**F**) neutrophil-to-lymphocyte (N/L) ratio stratified by dominant genotype model (CC vs. CT + TT) among PLWH on ART with metabolic syndrome and control group. Boxes represent interquartile ranges (IQR), horizontal lines indicate medians, and whiskers represent minimum and maximum values. P-values were calculated using the Mann–Whitney U test.

Further analyses stratified by MS status is presented on Fig 4. Among PLWH with MS, carriers of the CT + TT genotype had significantly elevated IL-6 levels compared to CC genotype carriers (p < 0.05). No other significant differences in inflammatory markers were observed between genotypes.

### 3.7. Multivariable logistic regression analysis of CRP and fibrinogen in MS

A multivariable logistic regression analysis identify several independent predictors of MS (Table 3). Elevated log-transformed CRP levels were independently associated with MS (OR 15.41, 95% CI 1.42–167.25; p = 0.025). Age was also independently associated with MS (OR 1.12 per year, 95% CI 1.05–1.19; p = 0.001). Fibrinogen, analyzed in tertiles, showed increased odds of MS in the middle tertile compared to the lowest tertile, while no significant difference was observed for the highest tertile. The *HIF1α* rs11549465 CT + TT genotype was associated with reduced odds of MS (OR 0.20, 95% CI 0.07–0.60; p = 0.004). No significant associations were observed for ART duration or ART regimen group. The model demonstrated good fit (Hosmer–Lemeshow p = 0.185) and explained a substantial proportion of variance (Nagelkerke R² = 0.52).

**Table 3:** Final logistic regression model predicting the presence of MS.

Variables	B	S.E.	Wald	*p*	OR	95% CI
LogCRP	2.735	1.217	5.053	**0.025**	15.41	1.42 - 167.25
Age	0.109	0.033	10.865	**0.001**	1.12	1.05 - 1.19
ART duration	0.004	0.046	0.007	0.933	1.00	0.92 - 1.10
Fibrinogen (tercile)			5.533	0.063		
Fibrinogen T2vsT1	1.611	0.685	5.532	**0.019**	5.01	1.31 - 19.16
Fibrinogen T3vsT1	1.026	0.714	2.062	0.151	2.79	0.69 - 11.31
ART overall			0.386	0.824		
PI based group^†^	0.502	0.930	0.291	0.589	1.65	0.27 - 10.23
INSTI based group^†^	0.400	0.692	0.334	0.563	1.49	0.38 - 5.80
rs11549465^‖^	−1.601	0.552	8.416	**0.004**	0.20	0.07 - 0.60
Constant	−7.454	1.652	20.372	0.000	0.00	

Statistically significant results are shown in bold. Abbreviations: logCRP – log-transformed C-reactive protein; T1 - Tercile 1; T2 - Tercile 2; T3 - Tercile 3; ART – Antiretroviral therapy; PI – Protease inhibitors; INSTI – Integrase Strand Transfer Inhibitors; B – the regression coefficient; S.E. - the standard error; Wald χ2 – Wald test statistics for the degree of freedom of 1 (df = 1); p – the probability; OR – Odds ratio; 95% CI – the 95% confidence interval for the estimated OR.† - NNRTI as reference category; ‖ - dominant genotype, CC as reference category.

## 4. Discussion

Despite substantial improvements in survival, quality of life, and viral suppression with ART, treated HIV remains characterized by persistent low-grade inflammation [22]. MS and related comorbidities are increasingly common in PLWH, partly due to aging, longer HIV duration, and cumulative ART exposure [2,23–26]. Consistently, MS in our cohort was more frequent among older participants with longer HIV infection and ART exposure. This may reflect “inflammaging” whereby aging and HIV-related immune activation creates a pro-inflammatory environment that promotes cardiometabolic complications, as also suggested by Prakoeswa et al. [27].

The heterogeneity of antiretroviral regimens and changes in therapy during follow-up limited the feasibility of detailed subgroup analyses based on ART. In unadjusted analyses, the prevalence of MS differed across current ART regimens, being highest in participants receiving PI-based therapy (53.3%), intermediate in those on INSTI-based regimens (40.5%), and lowest in the NNRTI group (22.7%). Although PIs remain widely used in HIV treatment, their use has been associated with adverse metabolic outcomes, including MS, leading to their decreased usage in recent years [28]. This trend, along with the rising preference for INSTI, is corroborated by the findings of this study. Recent research indicates that INSTIs, such as dolutegravir and bictegravir, might also be linked to weight gain and certain elements of MS in PLWH [29]. The ADVANCE and NAMSAL trials reported higher incidences of MS with dolutegravir compared to efavirenz, particularly when dolutegravir was used alongside tenofovir alafenamide [30]. Additionally, large cohort studies have associated INSTI use with the onset of diabetes [31,32]. It is important to interpret these findings with caution, as factors such as age, initial body fat, sex, the return-to-health effect, and the accompanying NRTI backbone may also affect metabolic outcomes. However, the connection between INSTI treatment, inflammation, and MS in individuals with HIV is not yet clearly understood. A study conducted in Zambia found that both dolutegravir-based treatment and elevated hs-CRP levels were independently linked to MS [33]. Thus, INSTI use is likely one of several contributors to metabolic risk rather than a sole cause of MS.

The duration of HIV infection and ART exposure have shown inconsistent associations with low-grade inflammation. Most studies report positive associations with hs-CRP and IL-6 [34–36], whereas Bello et al. found inverse associations, possibly due to differences in disease stage, immunosuppression, ART regimen, and comorbidities [37]. In our study, no significant associations were observed between these factors and inflammatory markers.

Our results showed significantly higher values of CRP and fibrinogen in PLWH with MS, which is consistent with findings from previous studies. Numerous studies in the general population have demonstrated elevated CRP and fibrinogen levels in individuals with MS compared to healthy controls [15,38,39]. In PLWH, available data are more limited. Duro et al. reported higher levels of hs-CRP and IL-6 in PLWH with MS [40], while Appiah et al. demonstrated an association between MS and elevated hs-CRP levels in this population [34]. Recent research in the PLWH has focused on determining markers of chronic inflammation in the context of atherosclerosis, major cardiovascular events, and aging, and not comprehensively in the context of the MS. In these studies, hsCRP has a confirmed prognostic value, but it is not HIV specific and depends on other factors [41–43], while other biomarkers, especially IL-6, D-dimer and markers of monocyte activation, confirm the pattern of higher inflammatory activation in PLWH [41–43]. Additionally, there are only few recent studies that have examined hsCRP or CRP concentrations in PLWH with MS, and most of them have not conducted a direct, adjusted analysis of the association [44,45].

Our results showed that CRP levels increased with age which is consistent with data from the general population [46,47]. Similarly, data from the Multicenter AIDS Cohort Study (MACS) demonstrate that CRP levels increase with age in PLWH, regardless of viral suppression status, and consistently exceed those observed in HIV-negative men, suggesting that HIV-related inflammaging further amplifies the inflammatory burden associated with MS [48,49]. In our study, the association was demonstrated using standard CRP measurements. Previous studies have shown that standard CRP and hs-CRP provide comparable results, supporting the use of standard CRP as an acceptable alternative for assessing low-grade inflammation [17]. An important advantage of standard CRP over hs-CRP is its wider availability and lower cost, which is particularly relevant in our study. Improved sensitivity of standard CRP assays may explain their comparability with hs-CRP in assessing CVD risk [50]. However, in accordance with current guidelines, the lack of hs-CRP measurement represents a limitation of our study [51].

Recent studies in PLWH have examined fibrinogen mainly in relation to coagulation, renal function, and cardiovascular risk rather than MS, leaving an important evidence gap, which gives additional significance to the results presented in our research. Nevertheless, elevated fibrinogen during ART is well documented and may reflect persistent immune and coagulation activation [7,52]. In our cohort, fibrinogen was higher in PLWH with MS, consistent with its reported associations with HTA, hypertriglyceridemia, and low HDL-C in both the general population and HIV-infected individuals. Reduced HDL-C impairs anti-inflammatory and antithrombotic protection, thereby contributing fibrinogen-associated vascular dysfunction [53,54]. These metabolic abnormalities may promote inflammation, thrombosis, and endothelial dysfunction, further intensified in PLWH by persistent immune activation and ART-related changes in adipose tissue and lipid metabolism, leading to an increased cardiometabolic risk profile. [8,55].

Although median CRP and fibrinogen levels were higher in several MS-related subgroups, these associations were attenuated when ROC-derived cut-off values were applied. This is expected, as the cut-off values were optimized to predict MS as a composite outcome rather than its individual components. Dichotomization of continuous biomarkers reduces statistical power and may conceal associations that are detectable when variables are analyzed on a continuous scale. This effect is especially strong in PLWH, where CRP and fibrinogen levels show substantial variability due to persistent low-grade inflammation and ART-related metabolic effects. Consequently, analysis based on continuous variables exhibited greater sensitivity in detecting subgroup differences compared to those based on binary classifications.

The correlations between CRP, fibrinogen, and IL-6 reflect a persistent pattern of low-grade systemic inflammation, characterized by concurrent elevations in these markers. Such associations are expected, considering that IL-6 is a principal upstream regulator of the acute-phase response and directly facilitates hepatic synthesis of both CRP and fibrinogen, corroborating previous findings that designate IL-6 as a central mediator of systemic inflammation [56]. In our ART-treated PLWH cohort, ferritin and D-dimer were not significantly associated with MS or its associated comorbidities. Although IL-6 is widely recognized as a major inflammatory mediator in PLWH, particularly in viremic or immunologically unstable populations, its levels did not differ significantly between participants with and without MS in our study [57]. The reason probably lies in the low level of inflammation in our long-term ART-suppressed population, the observed narrow IL-6 range, and the possible anti-inflammatory effects of concomitant therapies such as statins or antihypertensives [58–60]. To our knowledge, no previous studies in PLWH have established ROC-based cut-off values for CRP or fibrinogen specifically for MS. Most studies in HIV population use standard hs-CRP risk categories (e.g., > 1 mg/L or >3 mg/L), derived from general CVD risk guidelines, without validation for MS in this specific group [49,61,62]. In the general population, fibrinogen has been associated with MS and cardiometabolic risk, with higher levels (approximately 3.0–3.8 g/L) considered clinically relevant, particularly in individuals with type 2 DM or vascular complications [63–65]. In contrast, our ROC analysis in ART-treated PLWH demonstrated lower optimal cut-off values for both CRP (1.85 mg/L) and fibrinogen (2.49 g/L), suggesting that clinically relevant inflammatory and prothrombotic processes may occur at lower biomarker levels in this population. Our results may have clinical implementation, as CRP and fibrinogen could complement existing MS criteria by identifying PLWH at increased risk of CVD and DM type 2. Although the ROC-derived cut-off values for CRP and fibrinogen demonstrated only modest discriminatory ability, they showed statistically significant association and potential usefulness for risk stratification rather than diagnostic purposes. Importantly, these thresholds were derived to predict MS as a composite outcome and should be considered as population-specific and hypothesis-generating. Their clinical applicability require validation in independent cohorts, and at present, they should be interpreted as adjunctive markers in research setting.

Large genetic databases, such as gnomAD, report that the minor T allele frequency of the rs11549465 polymorphism is approximately 10.1% in Europeans, with TT homozygotes accounting for about 0.5–1% of individuals [66]. The role of *HIF-1α* gene SNPs, specifically rs11549465, in MS and related conditions has been more extensively studied in the general population, where most results suggest a protective effect of the minor T allele, consistent with our findings, although conflicting data exist [67–69]. In contrast, studies in PLWH remain limited. *HIF-1α* has been linked to certain parameters of MS including HTA, obesity, or dysregulated glycemia [15]. Given that chronic inflammation underlies MS pathogenesis, and that persistent immune activation is a hallmark of HIV infection and a key driver of non-AIDS comorbidities, the association of HIF-1α and metabolic and inflammatory pathways in PLWH is of particular interest. Mechanistically, HIF-1α contributes to HIV-associated inflammation by stimulating the release of extracellular vesicles that trigger the secretion of numerous inflammatory mediators [70]. The rs11549465 (Pro582Ser) variant is a functional polymorphism affecting HIF-1α stability and transcriptional activity [16,71,72], and has been linked to obesity, DM, and CVD, potentially through enhanced hypoxia-driven inflammatory signaling and HIF-1α–NF-κB interaction [73–75]. In this mechanistic context, our finding of an rs11549465–IL-6 association in ART-treated PLWH with MS may signify the initial evidence of this genotype–phenotype interaction in this cohort, aligning with established HIF-1α–IL-6 regulatory pathways. These findings are in line with our previous study in a similar cohort, where the *HIF-1α* rs11549465 polymorphism was associated with metabolic syndrome, further supporting its role in metabolic and inflammatory pathways in PLWH [19].

This study has some limitations. The relatively small sample size may have reduced statistical power and potentially may have increased the possibility of Type II errors. Therefore, the ROC-derived thresholds, subgroup findings, and multivariable results should be interpreted with appropriate caution and validated in larger independent cohorts. In addition, the observational design precludes causal inference, and the findings may be specific to the studied population of PLWH. Hs-CRP was not measured due to technical constrains. Data on lifestyle-related inflammatory factors were not available. Furthermore, other potentially important *HIF-1α* variations were not evaluated and may have influenced the observed associations. Finally, the participants were only classified using the NCEP ATP III criteria, so the prevalence estimates could change if different definitions of MS were used.

## 5. Conclusion

Our results suggest that CRP and fibrinogen can complement existing MS criteria to better identify PLWH at risk for cardiovascular disease and diabetes. The selective association of *HIF-1α* rs11549465 polymorphism with IL-6 may reflect IL-6-specific hypoxic and adipose-tissue signaling, whereas CRP and fibrinogen may be influenced by broader genetic, metabolic, and environmental factors. By being among the first to propose specific CRP and fibrinogen cut-off values for MS in PLWH, this study advances the understanding of the hypoxia-inflammation axis. Larger cohort studies are needed to validate these findings and confirm their clinical applicability.
